# Supplementary material for: Psychometric Properties of the Identity Bubble Reinforcement Scale (IBRS) in a Sample of Chilean Adolescent Students
Source: Children (Basel). 2025 Nov 14;12(11):1545. doi: 10.3390/children12111545 (PMC12651286; doi:10.3390/children12111545)
Supplement: Supplementary file 1 [file children-12-01545-s001.zip › children-3922074-supplementary.pdf]

Supplementary materials

The following supplementary materials are provided to accompany our manuscript:

Table S1. Items in the original English version of IBRS-9 and the version in Spanish.

Instrucciones/ **Instructions:**

A continuación, encontrarás cinco afirmaciones con las que puedes estar totalmente de acuerdo o totalmente en desacuerdo. Indica tu grado de acuerdo con cada afirmación / **Below, you will find five statements with which you may either totally agree or totally disagree. Please indicate your level of agreement with each statement.**

Versión original en español / Original version in Spanish

		Totalmente en desacuerdo	En desacuerdo	Indiferente	De acuerdo	Totalmente de acuerdo
1	En las redes sociales, pertenezco a una comunidad/es o grupo/s que son parte importante de mi identidad.	☐	☐	☐	☐	☐
2	En las redes sociales, pertenezco a una comunidad/es o grupo/s de las cuales me siento orgulloso/a.	☐	☐	☐	☐	☐
3	En las redes sociales, prefiero interactuar con personas parecidas a mí.	☐	☐	☐	☐	☐
4	En las redes sociales, prefiero interactuar con personas que comparten intereses similares a los míos.	☐	☐	☐	☐	☐
5	En las redes sociales, confío en la información que comparten conmigo.	☐	☐	☐	☐	☐
6	En las redes sociales, siento que la gente piensa como yo.	☐	☐	☐	☐	☐
7	En las redes sociales, pertenezco a una comunidad/es o grupo/s con los que me puedo comprometer.	☐	☐	☐	☐	☐
8	En las redes sociales, prefiero interactuar con personas que comparten mis valores.	☐	☐	☐	☐	☐
9	En las redes sociales, me puedo mantener informado/a.	☐	☐	☐	☐	☐

Versión en inglés / English version

		Strongly Disagree	Disagree	Indifferent	Agree	Strongly Agree
1	In social media, I belong to a community or communities that are important part of my identity.	☐	☐	☐	☐	☐
2	In social media, I belong to a community or communities that I'm proud of.	☐	☐	☐	☐	☐
3	In social media, I prefer interacting with people who are like me.	☐	☐	☐	☐	☐
4	In social media, I prefer interacting with people who share similar interests with me.	☐	☐	☐	☐	☐
5	In social media, I trust the information that is shared with me.	☐	☐	☐	☐	☐
6	In social media, I feel that people think like me.	☐	☐	☐	☐	☐
7	In social media, I belong to a community or communities that I can commit to.	☐	☐	☐	☐	☐
8	In social media, I prefer interacting with people who share my values.	☐	☐	☐	☐	☐
9	In social media, I can keep myself well informed.	☐	☐	☐	☐	☐

Table S2. Standardized Factor Loadings, Factor Correlations, and Reliability Indices for the Three-Factor Correlated Model of the IBRS-9

**Factor / Indicator**	**Estimate**	**S.E.**	**Est./S.E.**	***p*-Value**
**Social Identity**				
Y1	0.893	0.007	119.211	< .001
Y2	0.904	0.006	149.873	< .001
Y7	0.807	0.009	90.454	< .001
**Homophily**				
Y3	0.871	0.008	106.460	< .001
Y4	0.889	0.010	84.926	< .001
Y8	0.750	0.011	68.385	< .001
**Confirmation Bias**				
Y5	0.592	0.019	31.190	< .001
Y6	0.635	0.019	33.619	< .001
Y9	0.468	0.017	27.934	< .001
Reliability indices				
Omega total (ω_t)	0.78	—	—	—
Average Variance Extracted (AVE)	0.63	—	—	—

Note: Estimate = standardized loading; S.E. = standard error; Est./S.E. = critical ratio; p-Value = two-tailed significance level.

Table S3. Standardized Factor Loadings, Factor Correlations, and Reliability Indices for the Three-Factor Correlated Model of the IBRS-6

**Factor / Indicator**	**Estimate**	**S.E.**	**Est./S.E.**	***p*-Value**
**Social Identity**				
Y1	0.902	0.012	77.693	< .001
Y2	0.912	0.012	73.483	< .001
**Homophily**				
Y3	0.900	0.015	60.931	< .001
Y4	0.890	0.017	51.121	< .001
**Confirmation Bias**				
Y5	0.618	0.025	25.127	< .001
Y6	0.738	0.019	38.527	< .001
**Reliability indices**				
Omega total (ω_t)	**0.67**	—	—	—
Average Variance Extracted (AVE)	**0.60**	—	—	—

Note: Estimate = standardized loading; S.E. = standard error; Est./S.E. = critical ratio; p-Value = two-tailed significance level.

Table S4. Standardized Factor Loadings and Reliability Indices for the Second-Order Confirmatory Factor Model of the IBRS-9.

**Factor / Indicator**	**Estimate**	**S.E.**	**Est./S.E.**	***p*-Value**
**Social Identity**				
Y1	0.844	0.006	143.540	< .001
Y2	0.929	0.007	128.116	< .001
Y7	0.818	0.009	87.018	< .001
**Homophily**				
Y3	0.870	0.008	106.790	< .001
Y4	0.889	0.010	85.590	< .001
Y8	0.751	0.011	68.348	< .001
**Confirmation Bias**				
Y5	0.593	0.019	30.589	< .001
Y6	0.628	0.019	33.817	< .001
Y9	0.473	0.017	28.211	< .001
**Second order (G)**				
**Social Identity**	0.636	0.015	41.355	< .001
**Homophily**	0.825	0.015	54.650	< .001
**Confirmation Bias**	0.990	0.024	42.126	< .001
**Reliability indices**				
Omega hierarchical (ω_h)	**0.52**	—	—	—
Omega total (ω_t)	**0.74**	—	—	—

Note: Estimate = standardized loading; S.E. = standard error; Est./S.E. = critical ratio; p-Value = two-tailed significance level.

Table S5. Standardized Factor Loadings and Reliability Indices for the Second-Order Confirmatory Factor Model of the IBRS-6.

Factor / Indicator	Estimate	S.E.	Est./S.E.	*p*-Value
**Social Identity**				
Y1	0.844	0.008	105.982	< .001
Y2	0.970	0.012	81.716	< .001
**Homophily**				
Y3	0.899	0.015	60.648	< .001
Y4	0.890	0.017	51.125	< .001
**Confirmation Bias**				
Y5	0.618	0.025	24.855	< .001
Y6	0.738	0.019	38.676	< .001
**Second order (G)**				
Identidad	0.636	0.021	30.513	< .001
Homofilia	0.746	0.017	43.748	< .001
**Confirmation Bias**	0.865	0.022	39.151	< .001
**Reliability indices**				
Omega hierarchical (ω_h)	**0.45**	—	—	—
Omega total (ω_t)	**0.62**	—	—	—

Note: Estimate = standardized loading; S.E. = standard error; Est./S.E. = critical ratio; p-Value = two-tailed significance level.

Table S6. Heterotrait–Monotrait (HTMT) Ratios Between Latent Factors of the IBRS-9 (Three-Factor Correlated Model).

Latent Factor	Social Identity	Homophily	Confirmation Bias
**Social Identity**	—	**0.54**	**0.67**
**Homophily**	0.54	—	**0.79**
**Confirmation Bias**	0.67	0.79	—

Note. HTMT = Heterotrait–Monotrait ratio of correlations; IBRS-9 = Identity Bubble Reinforcement Scale, nine-item version.

Table S7. Heterotrait–Monotrait (HTMT) Ratios Between Latent Factors of the IBRS-6 (Three-Factor Correlated Model).

Latent Factor	Social Identity	Homophily	Confirmation Bias
**Social Identity**	—	**0.48**	**0.57**
**Homophily**	0.48	—	**0.64**
**Confirmation Bias**	0.57	0.64	—

Note. HTMT = Heterotrait–Monotrait ratio of correlations; IBRS-6 = Identity Bubble Reinforcement Scale, six-item version.
